# The magnitude of severe box jellyfish cases on Koh Samui and Koh Pha-ngan in the Gulf of Thailand

**DOI:** 10.1186/s13104-016-1931-8

**Published:** 2016-02-17

**Authors:** Lakkana Thaikruea, Potjaman Siriariyaporn

**Affiliations:** Department of Community Medicine, Faculty of Medicine, Chiang Mai University, Chiang Mai, 50200 Thailand; Epidemiology Bureau, Department of Disease Control, Ministry of Public Health, Nonthaburi, 10100 Thailand

**Keywords:** Box jellyfish, Toxin, Thailand, Fatality, Envenomation, Island, Cubozoan

## Abstract

**Background:**

Despite recent deaths caused by box jellyfish envenomation occurring on the islands of Samui and Pha-ngan in the Gulf of Thailand, many people do not believe box jellyfish can kill humans and many people dismiss the problem as insignificant. More evidence has been requested from the communities in order to evaluate the need for and the implementation of sustainable prevention measures. We aimed to determine the magnitude of cases of severe stinging by box jellyfish and describe the characteristics of these cases on the islands of Samui and Pha-ngan in Surat Thani Province from 1997 to 2015.

**Methods:**

Various strategies were integrated prospectively. Toxic jellyfish networks and surveillance system were established. Outbreak investigations were conducted retrospectively and prospectively from 2008 to 2015.

**Results:**

There were 15 box jellyfish cases. A small majority of them were women (60.0) with a median age of 26.0 years (range 5.0–45.0 years). The highest incidence by month were August (33.3 %), September and October (20.0 %), and July (13.3 %). Eight cases occurred on Samui (53.3 %), 6 cases on Pha-ngan island (40.0 %), and one case on the boat. All cases developed symptoms and signs immediately after being stung. More than half of the cases were unconscious. There were six fatal cases (46.7 %). The wound characteristics had an appearance similar to caterpillar tracks or step ladder-like burn marks. Almost all cases involved Chirodropidae. One fatal case received fresh water and ice packs applied to the wounds (16.7 %). Among the cases with known first aid, only one out of six fatal cases had vinegar applied to the wounds (16.7 %), while haft of six surviving cases received the vinegar treatment.

**Conclusions:**

The islands of Samui and Pha-ngan have the highest incidence of fatal and near fatal box jellyfish cases in Thailand. There is an urgent need for informed pre-clinical emergent care. Optimal pre-clinical care is an area of active research.

## Background

It is widely known that box jellyfish are one of the most venomous marine animals in the world [1]. Envenomation involves the physical discharge of venom into tissues. The venom is a complex mixture of polypeptides and proteins, including hemolytic, cardiotoxic and dermatonecrotic toxins [2–4]. There are two major families of box jellyfish, are Chirodropidae and Carybdeidae [5, 6]. The most lethal member of Chirodropidae is *Chironex fleckeri*. Envenomation by Chironex can lead to “rapid cardiorepsiratiory depression”. The injured person who received a high dose of toxin can die within a few minutes [2, 4, 5, 7–10], Local physicians, nurses and other health personnel still lack the necessary knowledge regarding box jellyfish, despite there being reports of envenomation incidents in Thailand. Thus, diagnosis of Irukandji syndrome (often associated with carybdeid stings) and other box jellyfish envenomation sequelae has been rare in Thailand [1]. According to Thaikruea et al. [11] a study of the morbidity and mortality rates of toxic jellyfish stings from thirty-three health services in the southern provinces of Thailand (Surat Thani, Krabi, Phuket, and Satun), there were 381 cases involving toxic jellyfish between 2003 and 2009. However, only 51 cases were diagnosed as being in the toxic jellyfish category, one case of neuropathy from the neurotoxin of a jellyfish, and one case of suspected Irukandji syndrome. While the Thai surveillance system of toxic jellyfish injuries and deaths that was established in 2009 identified at least 38 toxic jellyfish cases within 1 year period, Thaikruea et al. [12] reported at least four fatal and four near fatal probable box jellyfish cases in Thailand. Three out of four fatal cases occurred on the islands of Pha-ngan and Samui in the Surat Thani province. These islands are located in the Gulf of Thailand. With the sparsity of information the magnitude of the problem is likely to be underestimated.

Proper first aid carried out at the scene is crucial for the survival of an injured individual who has been stung by a box jellyfish. Household, food grade vinegar (4–6 % acetic acid) is effective in acid fixation based inactivation of unfired nematocysts. It should be poured continuously on the wound for at least 30 s [5, 13]. One of the two latest deaths which was probably due to a box jellyfish sting was on July 31st 2015 and was quite naturally of great concern to the public (Nation TV. August 1st 2015. http://www.nationtv.tv/main/content/social/378466023/). This Thai woman aged 31 years was diagnosed as “Cardiac arrest with anaphylaxis following contact with a venomous animal”. One of the concerns about jellyfish envenomation is that a wrong diagnosis can lead to inappropriate treatment [14]. A marine biology expert recently announced in the press the need to use seawater splashing on the tentacle marks before pouring vinegar as a first aid treatment for box jellyfish stings. This news introduced confusion among both professionals and the public, in particular it has caused great concern for the people who live on the islands where the majority of the incidents have taken place. The local people tried to keep people safe. One protection measure used was the erection of warning signs saying “bluebottles” on the beaches that are famous for the “full moon parties”. This warning sign was wrong because the danger was ‘Box Jellyfish Species Present’. There are many cubozoan species linked to lethal stings. Both Chirodropids and Carybdeids can cause death. Signage should include images of both families of cubozoa and there was no education message written in the sign [15, 16].

The islands of Samui and Pha-ngan locate next to each other (20 km) in the Gulf of Thailand. A travel time is about 15 min by ferry. There are various conflicts of interest among stakeholders such as business owners, hotel/resort owners, fishermen, tourist guides and non-government organizations. Many people do not believe box jellyfish can injure or kill human based on interviews, meetings and workshops carried out over the past 7 years. Even of the people who accept the fact, many of them still think that the problem is small. More evidence is requested from the communities in order to implement sustainable prevention measures. This study aims to determine the magnitude of cases of severe stinging by box jellyfish and describe the characteristics of these cases on the islands of Samui and Pha-ngan in Surat Thani Province from 1997 to 2015.

**Fig. 1:**
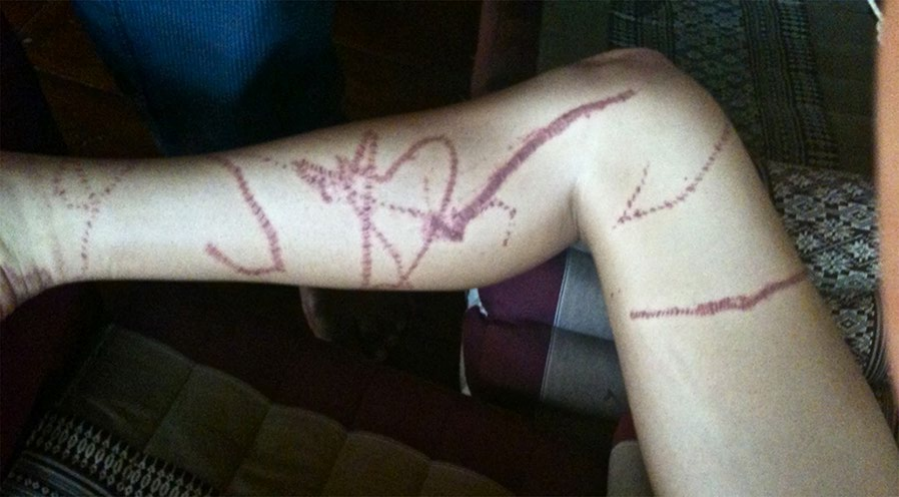
Near fatal case: a 26 year old American woman stung by a multiple tentacle box jellyfish on September 3rd 2010 at a beach on Pha-ngan island. She lost consciousness and revived following resuscitation. Vinegar was applied (Source photo: case)

## Methods

Various strategies were integrated prospectively. The strategies included establishing toxic jellyfish surveillance system, establishing toxic jellyfish networks, conducted case investigations, performed studies (about the situation in Thailand, existence of deadly box jellyfish, and first aid and treatment), participated and supported communities to create innovations (vinegar first aid pole and sting net), and implement prevention measures. The toxic jellyfish network was established in 2008 by this team of authors in order to ascertain whether box jellyfish could cause death in humans and whether this is a threat in Thailand. The initial members included a journalist and experts from universities in Australia and the Divers Alert Network (DAN). The membership expanded to stakeholders such as resort/hotel managers/owners, divers, speed boat/long-tail boat groups and biologists in order to gather more information (interviews and focus group discussion), build knowledge and collaborate regarding possible prevention programs. Toxic jellyfish surveillance was begun in 2009 to identify venomous jellyfish incidents, collect information regarding the cases and implement prevention measures. The team prospectively and retrospectively investigated all suspected cases box jellyfish sting outbreaks, which were detected by toxic surveillance system and networks. Only severe cases involving admission into the hospitals or health services in Samui and Pha-ngan islands between January 1997 and October 2015 were included in this study. Descriptive analysis was done, including proportion (%) and median (range: minimum to maximum).

The investigation was under the government service policy of emergency and public health problem. The ethics submission was not applicable. The descriptive analysis was done as group average and could not identify each individual. For participants who provided photos, we obtained consents to publish from them (or legal parent or guardian for children) to report individual patient data and photos.

## Results

### Demography

There were 15 cases of severe stinging by box jellyfish admitted to hospital from 1997 to 2015. The majority of them were women (60.0 %) and the median age was 26.0 years (minimum 5.0 years and maximum 45.0 years). The highest frequency of nationalities included British (20.0 %), American (13.3 %), German (13.3 %), and Italian (13.3 %). The other nationalities included French, Australian, Russian, Swiss, Chinese and Thai.

### Incidence by time

The highest incidence by year were 2012 (20.0 %), 2015 (20.0 %), 2014 (13.3 %) and 2002 (13.3 %). The highest incidence by month were August (33.3 %), September (20.0 %), October (20.0 %), and July (13.3 %). Two cases occurred in December and May.

### Incidence by place

Among eleven cases with information of incidence places, eight cases (53.3 %) occurred on Samui island and another six cases (40.0 %) occurred on Pha-ngan island. One case did not occur on the beach, the man took off his wet suit after diving and a tentacle attached to the wet suit stung his left elbow.

The highest number of cases occurred on East Rin beach of Pha-ngan island (27.3 %), Chawang beach of Samui island (27.3 %), and Bo Phut of Samui island (18.2 %). Two cases occurred on Lamai beach of Samui island and Khuat beach of Pha-ngan island, subsequently.

### Severity

**Fig. 2:**
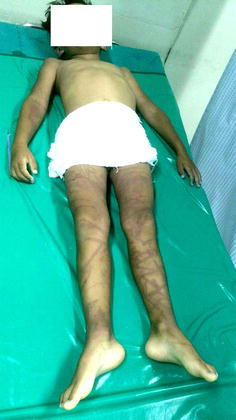
Fatal case: a 5 year old French boy, stung by a multiple tentacle box jellyfish on August 23rd 2014 at Khuat beach on Pha-ngan island. He lost consciousness, fresh water was poured over the injuries, ice packs were applied, and resuscitation was attempted by his parents (Source photo: parents of the boy)

**Fig. 3:**
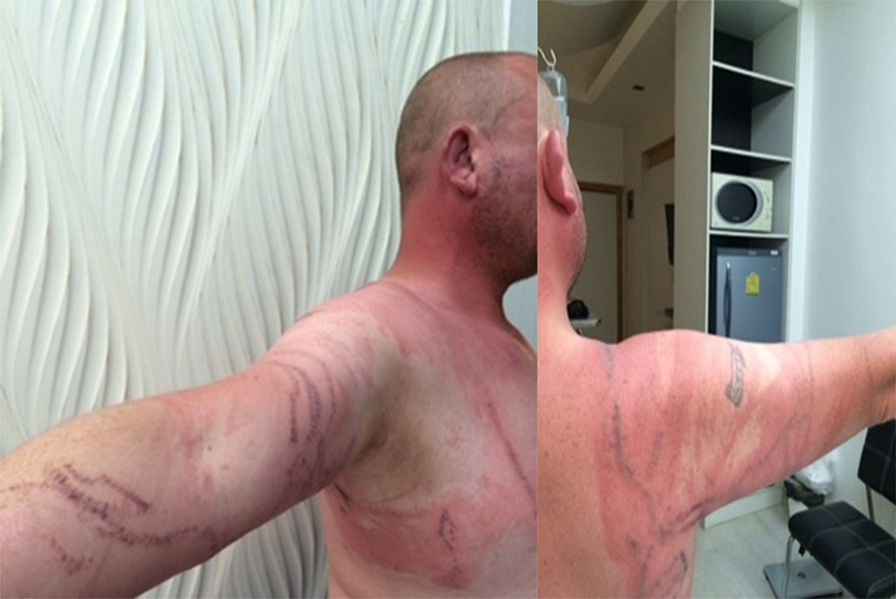
Near fatal case: a 45 year old British man, stung by a multiple tentacle box jellyfish on October 17th 2014 at Laem Som on Samui island. He had difficulty breathing and high heart rate. Vinegar was applied (Source photo: case)

**Fig. 4:**
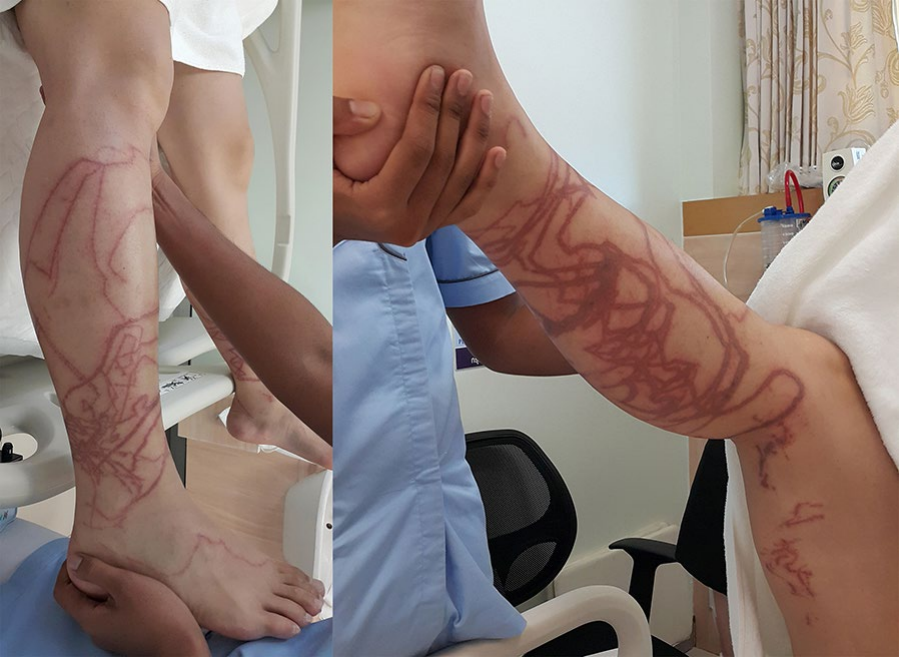
Near fatal case: a 31 year old Chinese man, stung by a multiple tentacle box jellyfish on September 12th 2015 at Chawang beach on Samui island. He lost consciousness and received vinegar and cardiopulmonary resuscitation at the hospital (10–15 min after stung). He was admitted into Intensive Care Unit and put on a respirator. (Source photo: hospital)

All cases developed symptoms and sign immediately within 1 min after being stung. More than half of the cases were unconscious (53.3 %) within 2–3 min. There were eight near-fatal cases (53.3 %), six fatal cases (46.7 %), and one case was discharged against advice (6.7 %). The wound characteristics had the appearance of caterpillar tracks or step ladder-like burn marks (Figs. 1, 2, 3, 4).

### Box jellyfish

Nine lethal jellyfish stings were reported as confirmed cubozoan stings. Of these, 5 or 55 % were reported as confirmed Chirodropid stings, three were reported as suspected Chirodropid stings and one case was due to a either a chirodropid or a carybdeid sting.

### First aid

Twelve cases were included in analysis where the first aid history was known. Of the six fatal cases, only one had vinegar poured on the injury (16.7 %) as first aid and one had fresh water poured on the wound followed by application of an ice pack (16.7 %). Among the six surviving cases, three received the vinegar treatment (50.0 %). One of them who was stung by Chirodropidae. He lost consciousness and was admitted to Intensive Care Unit, requiring use of a respirator. He received vinegar as first aid at the hospital after 10–15 min being stung (Fig. 4).

## Discussion

There were seven fatal box jellyfish cases in Thailand during the period 1999–2015. One death took place on Lanta island, Krabi Province. Six out of seven occurred on the islands of Pha-ngan and Samui which is the highest incidence of jellyfish related deaths in Thailand [11, 12, 14, 15]. This information is useful for convincing communities that the magnitude of the problem is high. Thai people and many local health personnel believe that the deaths may be due to a hypersensitivity of Caucasian people to jellyfish. A previous death of Swedish girl on March 2008 at a beach of Lanta Island, Krabi Province supports this belief. She was diagnosed as having anaphylactic shock from jellyfish contact. There were extensive tentacle marks all over her legs [12]. The nationalities of the probable box jellyfish cases in this study were varied. In addition to Caucasian people there were also Asian groups, including Chinese and Thai. In particular, the death of a Thai woman in this study was very high attention in the media and was of great public interest [14]. The diagnosis in this case was “Cardiac arrest with anaphylaxis following contact with a venomous animal”. Two of the reasons for misdiagnosis are lack of knowledge and misbelief. The authors with involving organizations have been gathering data to increase knowledge regarding box jellyfish in Thailand (Marine and Coastal Resources Research and Development Center of Marine and Coastal Resources, Department of Natural Resources and Environment Ministry, Surveillance and Rapid Response Teams, Regional Offices of Disease Prevention and Control National Science Museum, Phuket Marine Biology Center, Marine and Coastal Resources Research and Development Centers along the Gulf of Siam, government and private health services along the coasts of Thailand). However, information regarding various species of box jellyfish is not officially included the medical school curriculum yet. Nevertheless, some emergency medicine training courses and medical schools have started to include that knowledge from our text book [17]. The clinical manifestations of the female Thai victim were consistent with chirodropid envenomation. She collapsed and had no pulse within a few minutes. Her friends, who were also stung by the box jellyfish, removed the tentacles that were attached to her body and resuscitated her for about 20 min but there was no response. She was taken to a clinic nearby and received vinegar pouring and cardiopulmonary resuscitation (CPR). She did not respond and was transferred to a community hospital. After receiving CPR for an hour, she was declared dead at the community hospital. The time between being stung to receiving treatment by health personnel was about 20 min which was too long and she may well have died on the beach. Other cases in this study also had similar clinical manifestations which were indicative of chirodropid envenomations. One such case was the fatal sting of a German woman on October 6th, 2015. The injured individuals all suffered from tremendous pain after being stung and also had systemic reactions [2, 12]. They had difficulty breathing and had a very high heart rate. Thaikruea et al. [12] conducted the investigations and found that seven out of the eight cases collapsed and all of them had tentacle marks on their bodies.

Clinical observations support a dose dependency where envenomation severity is proportional to tentacle contact length. When the area of tentacle mark more than 50 % of extremity or the length of tentacle contact is more than 6–7 m the probability of death is high [2, 12]. In this study, most cases except the Australian male had wound areas of more than 50 % [11]. The remarkable signs were the wounds caused by the tentacles. From our observations, chirodropid envenomation associated dermatonecrosis differs from that caused by *Pelagia* spp. and *Chrysaora* spp. The brownish erythematous tentacle marks developed in a short period after being stung [12, 14, 16]. The wound was described by various professionals as “Frost-ladder like”, “Step ladder like”, “Ladder-like transverse band” or “whip marks” [5, 12, 14, 16, 18]. Thaikruea et al. [16] defined the term in Thai language as “caterpillar tracks” in order to educate the general population and health personnel in layman’s terms. The appearance of the wounds in the cases where the injuries were caused by Chirodropidae found in Thailand resemble the caterpillar tracks of the tanks which have articulated steel bands passing around the wheels. Sting site markings were typically 3–5 mm wide with approximate 1–2 mm long dermatonecrotic mark repeatedly interspersed by 1-3 mm of normal tissue. The length depend on tentacle contact lengths [12, 14, 16]. One great concern is that physicians who lack the relevant knowledge usually diagnose the box jellyfish stings as an allergic reaction or anaphylactic shock and treat the patients with antihistamine or steroid treatments. Steroids have no effect on dermatonecrotic toxin and may increase the probability of infection in the wounds. The treatment of the wound should be similar to those of fresh or burn wounds. Antibiotics can be used if necessary. In the later stages, steroids can be used if indicated [16].

The incidence of stings has increased in recent years. The higher incidence between July and October might be due to the seasonal life cycle of box jellyfish. The study of morbidity and mortality due to jellyfish envenomation from hospital data in the period from 1998 to 2002 indicated a high rate of incidence in the islands of Samui and Pha-ngan between August and October [19]. Another possible explanation is that this is high season for tourism on the islands. Logically, the more tourists there are on the islands, the more likely the contacts with jellyfish. The numbers of tourist are highest at the time of the full moon parties that are held each month on the night of the full moon on Rin beach, Pha-ngan island. In recent years, more parties have been occurring, including black moon and half moon parties. (http://www.fullmoonparty-thailand.com/; http://www.fullmoon.phangan.info/) These parties are of great concern to public health due to their popularity because many of the tourists become inebriated and swim in the dark sea. The beaches are very crowded and when incidents occur it is very difficult to recognize and rescue properly. It was on such an occasion that the latest death, that of the Thai female, occurred this year.

The majority of cases involved stinging by Chirodropidae. There are no experts or laboratory facilities to identify the toxin and species of box jellyfish in Thailand. The Epidemiology Bureau of Public Health Ministry established a collaborative agreement with the Faculty of Medicine, Chiang Mai University, Ministry of Education in 2008 [20]. The Australian experts have provided consultancy and the Marine and Coastal Resources Department of the Ministry of Natural Resources and Environment have joined the collaboration recently. Since then, the team has conducted outbreak investigations and studied the species of box jellyfish in Thailand. The latest results show that box jellyfish species may differ from those in Australia (Announcement in the meeting among key persons of stakeholder and government officers on Pha-ngan island on August 11, 2015).

Recently, there has been controversy about using vinegar for first aid to stop the firing nematocysts of box jellyfish when Seymour J from Queensland Tropical Health Alliance, School of Public Health and Tropical announced that vinegar induced the nematocysts to cause more venom to enter the body [21].Welfare et al. study only involved a battery stimulated tentacle, a placenta membrane and then various rinse solutions. There were no animal or human subjects. The results of this very small model was that vinegar led to a slight increase in recovery of very low (biologically irrelevant) levels of venom activity (at 1 μg/ml it was less than one hundredth of the native venom). The paper itself did not make wild claims, Jamie Seymour was quoted in the press releases as wildly exaggerating the findings to conclude that the small battery/membrane model indicated that “Vinegar can be deadly.” (Vinegar on jellyfish sting can be deadly: study. http://www.sbs.com.au/news/article/2014/04/08/vinegar-jellyfish-sting-can-be-deadly-study (accessed on 25 August 2015; Vinegar May Kill Rather than Cure Victims of Box Jellyfish Stings: Study. http://www.frenchtribune.com/teneur/1422205-vinegar-may-kill-rather-cure-victims-box-jellyfish-stings-study (accessed on 25 August 2015). These press release claims have not been supported by any other investigators and have been completely rejected by the entire scientific community. The study design and statistical validity has been criticized by Yanagihara and Chen [22]. Winkel K (director of the Australian Venom Research Unit, the University of Melbourne) expressed that there was not enough evidence to support this announcement. (http://www.cairnspost.com.au/news/cairns/scientists-salting-jcu-claims-vinegar-is-bad-for-jellyfish-stings/story-fnjpusyw-1226900575253) Ward et al. [23] concluded from their findings that vinegar made thing worse. However, Auerbach P disagreed because patients in the study received very small doses of vinegar, therefore, this conclusion was not valid. Furthermore, Auerbach P stated that based on his experiences in treating patients stung by box jellyfish he found that patients had good results when applying vinegar [15, 24]. Currently, the Australian Resuscitation Council and American Heart Association–American Red Cross International Consensus still recommend vinegar [13, 25]. Thaikruea et al. [12, 15, 20] found that the majority of near-fatal, or surviving cases in Thailand had vinegar poured onto the injuries as part of the first aid procedure, while the majority of the fatalities did not. In this study, only one of the fatal cases received vinegar as first aid but might be not appropriate. The fatality did not receive vinegar until 20 min after the sting. One fatal case in this study had fresh water poured on to the wounds and icepacks applied. It is now known that application of fresh water and ice cause greater firing of nematocysts, hence these treatments are contraindicated for first aid for box jellyfish stings [13, 25].

The incidents occurred more frequently in the southen part of Pha-ngan island which face the northern part of Samui island. However, the northern part and other areas also had incidents (data not shown for mild to moderate cases). Based on the experience of investigating the box jellyfish problem for this study on both Pha-ngan and Samui islands, it was found that people do not believe or do not know that the incidents are more prevalent than their perceptions. These perspectives were demonstrated at several meetings and workshops carried out over the past 7 years. After the death on July 31st this year, another severe case occurred on the island of Samui on September 12th 2015 at Chawang beach on Samui island. This occurred to a 31 year old Chinese male, who was stung by Chirodropidae. He lost consciousness and was admitted to Intensive Care Unit, requiring use of a respirator. He received vinegar as first aid at the hospital after 10–15 min being stung (Fig. 4). Stakeholders start to concern about this health problems and want to know more about the magnitude in these islands.

## Conclusions

The findings of this study are useful for providing scientific evidence of the magnitude of the box jellyfish stings and also for convincing the communities to take the problem seriously. Appropriate surveillance and sustainable prevention and control measures in all risk areas are in urgent need [20].

## Abbreviation

CPR: cardiopulmonary resuscitation
